# Supplementary material for: Trimetallic Chalcogenide Species: Synthesis, Structures, and Bonding
Source: Molecules. 2022 Nov 2;27(21):7473. doi: 10.3390/molecules27217473 (PMC9654038; doi:10.3390/molecules27217473)
Supplement: Supplementary file 1 [file molecules-27-07473-s001.zip › molecules-1929972-supplementary.pdf]

Trimetallic Chalcogenide Species: Synthesis, Structures, and Bonding

Sourav Kar [1,†], Debipada Chatterjee [1,†], Jean-François Halet[2,*] and Sundargopal Ghosh[1,*]

1 Department of Chemistry, Indian Institute of Technology Madras, Chennai 600036, India
2 Laboratory for Innovative Key Materials and Structures (LINK), IRL 3629, CNRS–Saint-Gobain–NIMS, National Institute for Materials Science (NIMS), Tsukuba 305-0044, Japan
* Correspondence: jean-francois.halet@univ-rennes1.fr (J.-F.H.); sghosh@iitm.ac.in (S.G.)
† These authors contributed equally to this work.

Table of Contents

I. Spectroscopic Details

Figure S1 ESI-MS of compound **1**

Figure S2 ^{1}H NMR spectrum of compound **1**

Figure S3 ^{11}B{^{1}H} NMR spectrum of compound **1**

Figure S4 ^{13}C{^{1}H} NMR spectrum of compound **1**

Figure S5 ESI-MS of compound **2**

Figure S6 ^{1}H NMR spectrum of compounds **2**

Figure S7 ^{11}B{^{1}H} NMR spectrum of compound **2**

Figure S8 ^{13}C{^{1}H} NMR spectrum of compounds **2**

Figure S9 Combined ESI-MS of compounds **3** and **4**

Figure S10 Combined ^{1}H NMR spectrum of compounds **3** and **4**

Figure S11 Combined ^{11}B{^{1}H} NMR spectrum of compounds **3** and **4**

Figure S12 Combined ^{13}C{^{1}H} NMR spectrum of compound **3** and **4**

II. Electronic Structure Analysis

Figure S13 Selected molecular orbitals of **1**

Figure S14 Selected molecular orbitals of **2**

Figure S15 Selected molecular orbitals of **3**

Figure S16 Selected molecular orbitals of **4**

Figure S17 Contour-line diagrams of the Laplacian of electron density in the S4-S5-S4' plane of **4**

Table S1 NICS values in compounds **1**-**4**

Table S2 Calculated natural charges (q_{Nb}, q_{B} and q_{E}), natural valence population (Pop) and HOMO–LUMO gaps of compounds **1**-**4** (E = Se or S)

Table S3 Wiberg Bond Indices (WBI) of selected bonds in **1**-**4**

Figure S18 Optimized geometry of **1**

Figure S19 Optimized geometry of **2**

Figure S20 Optimized geometry of **3**

Figure S21 Optimized geometry of **4**

I. Spectroscopic details

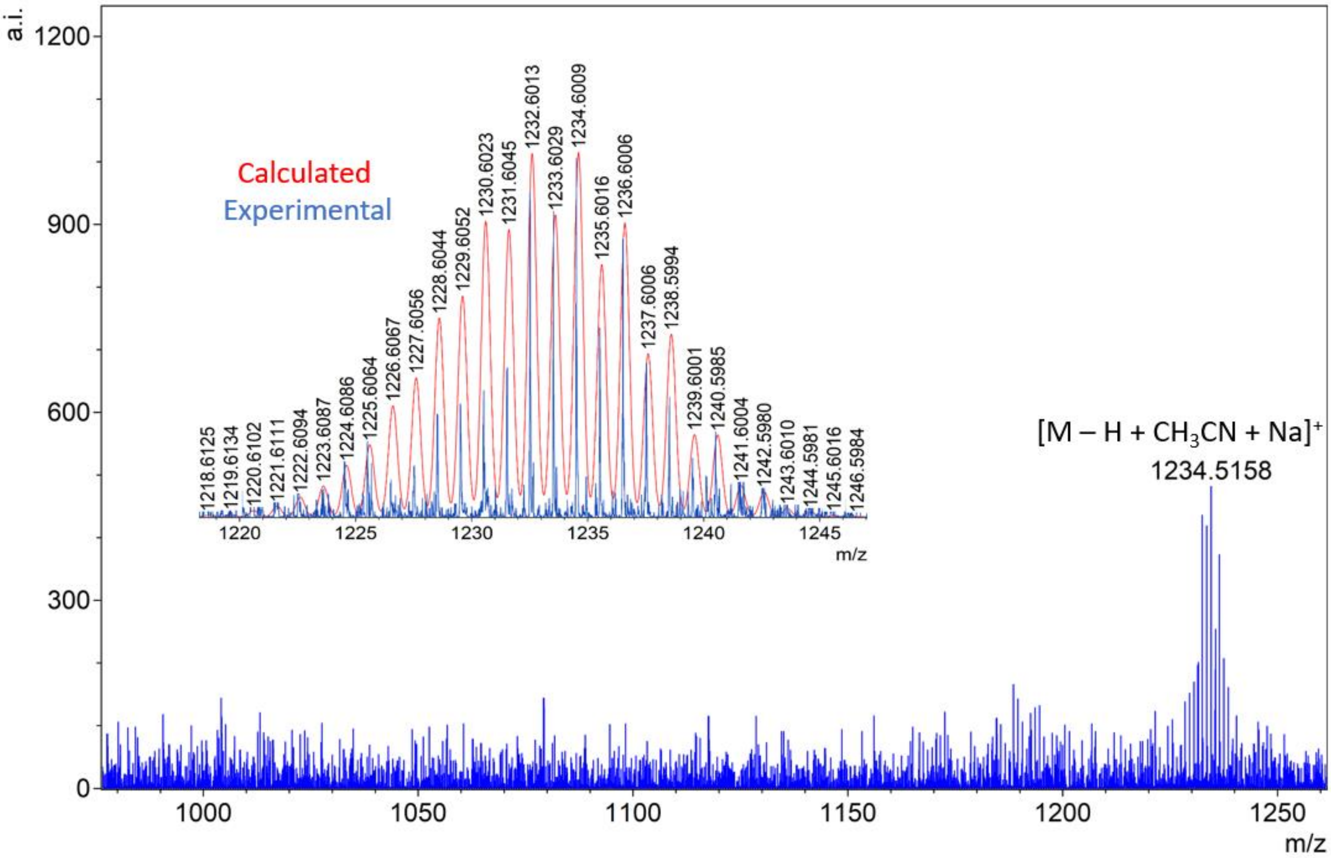

Figure S1. ESI-MS of compound **1**.

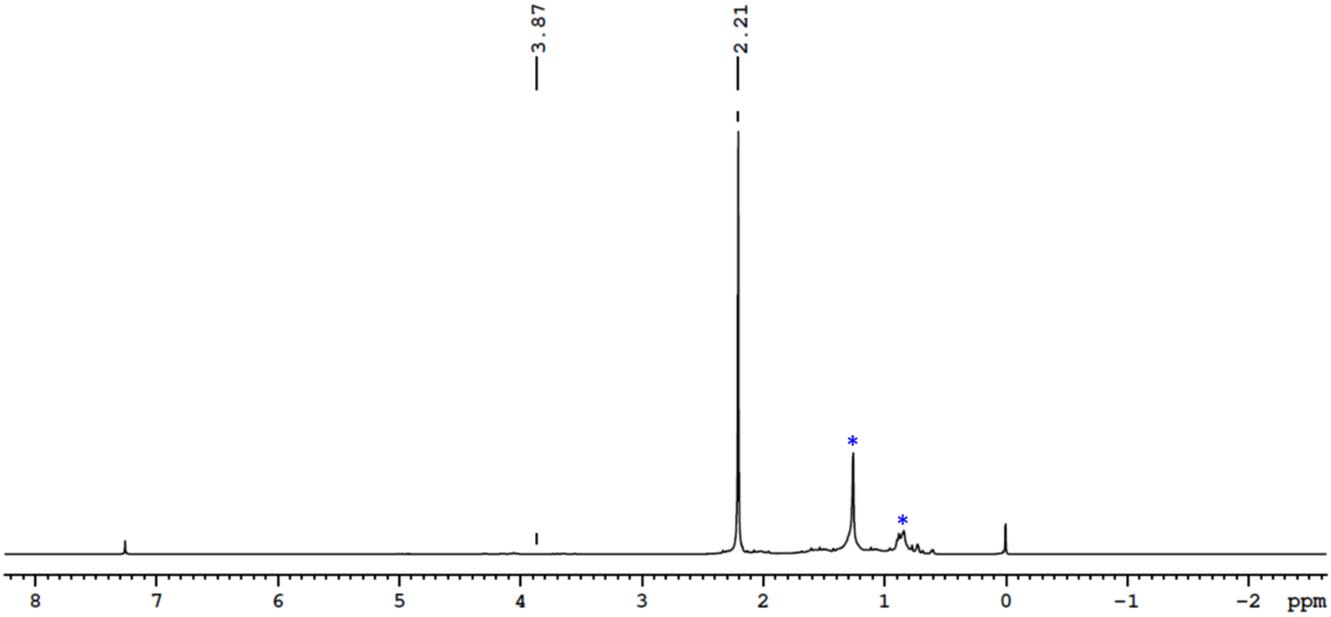

Figure S2. ^{1}H NMR spectrum of compound **1** in $CDCl_3$. (*Grease)

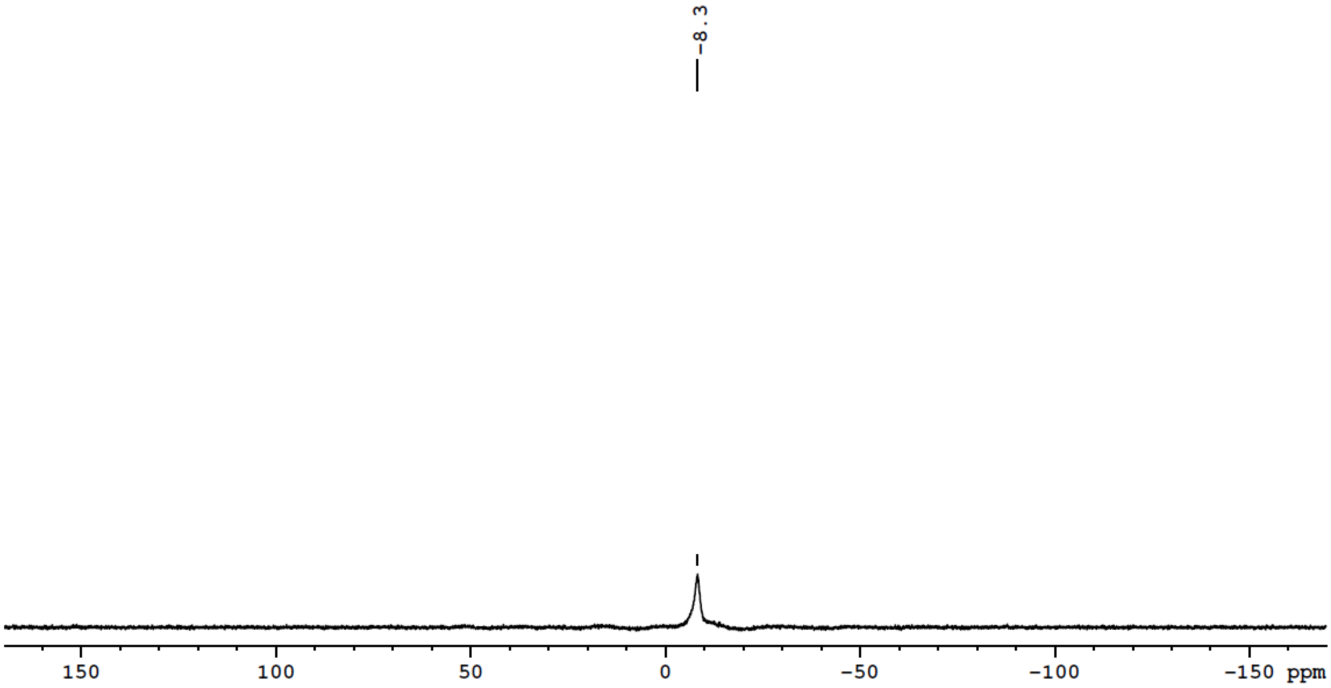

Figure S3. $^{11}B\{^{1}H\}$ NMR spectrum of compound **1** in $CDCl_3$ [1-4].

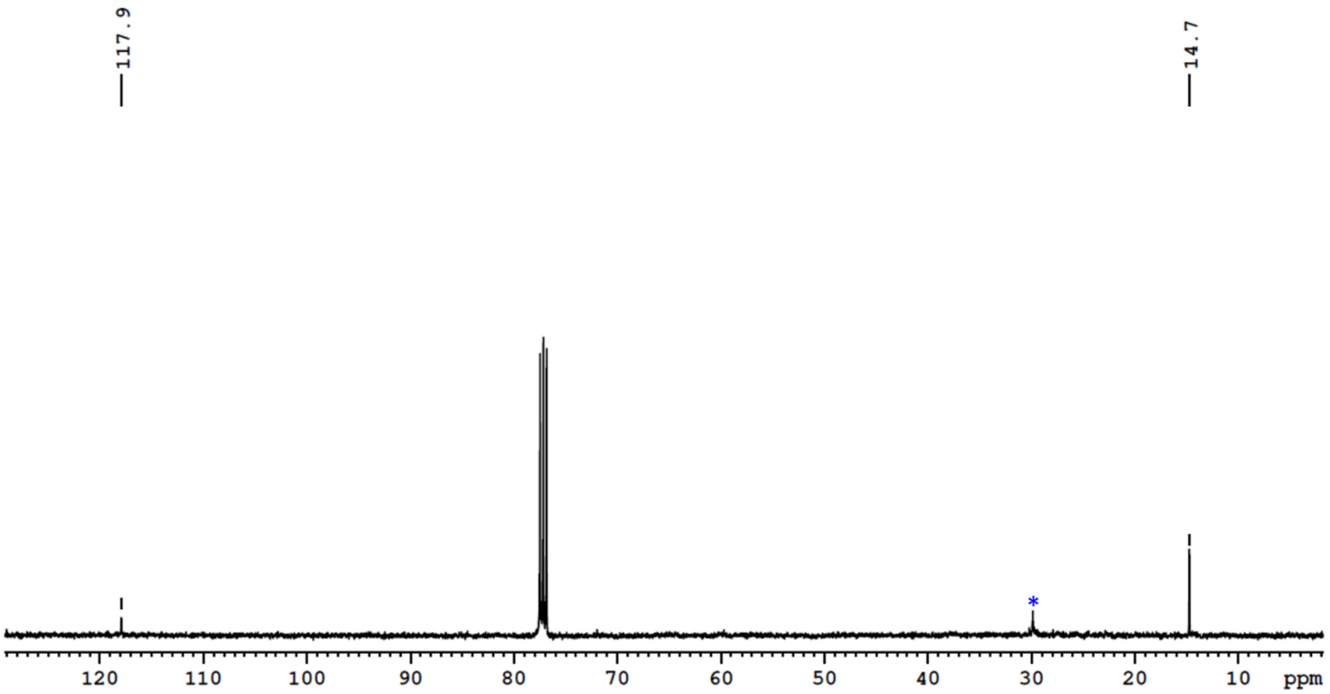

Figure S4. $^{13}C\{^{1}H\}$ NMR spectrum of compound **1** in $CDCl_3$. (*Grease)

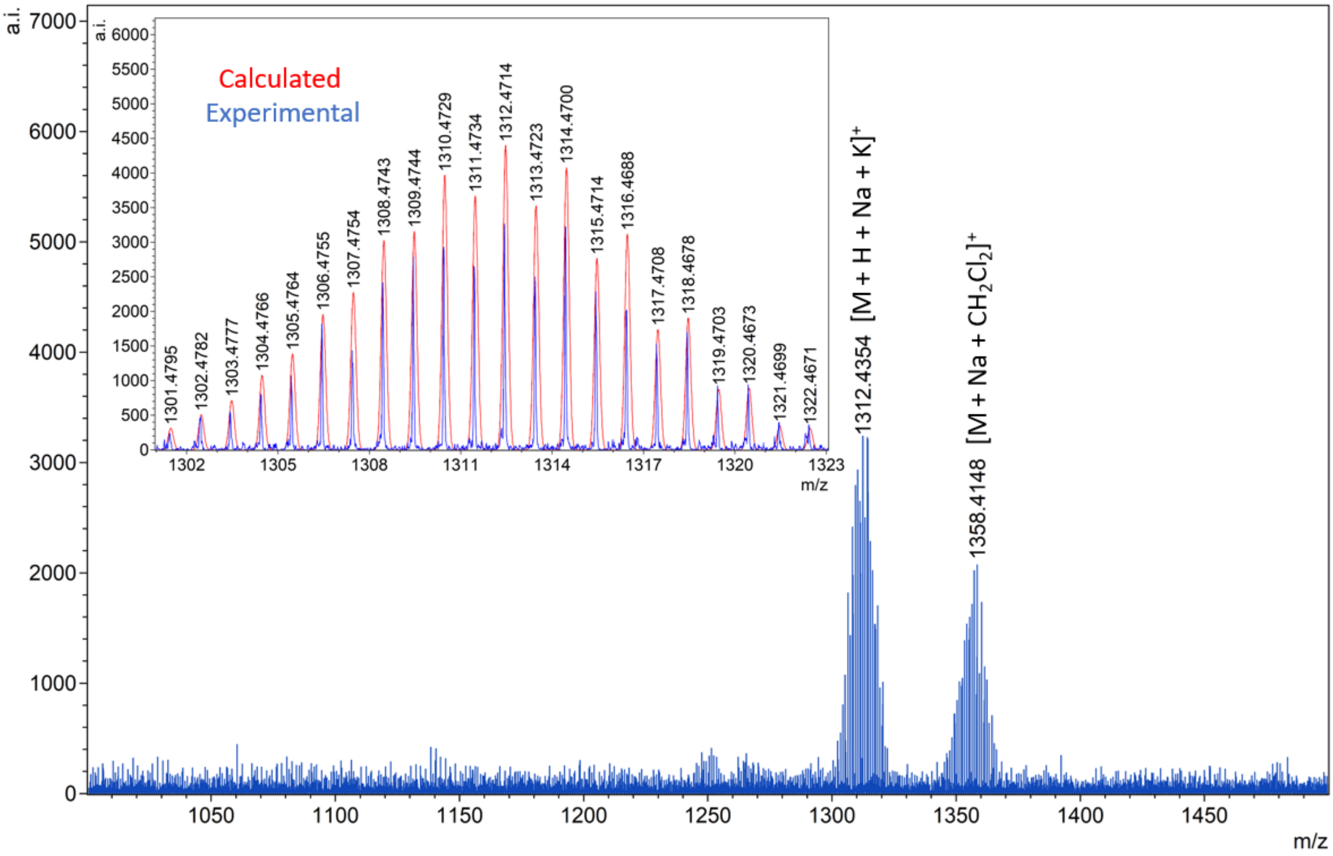

Figure S5. ESI-MS of compound **2**.

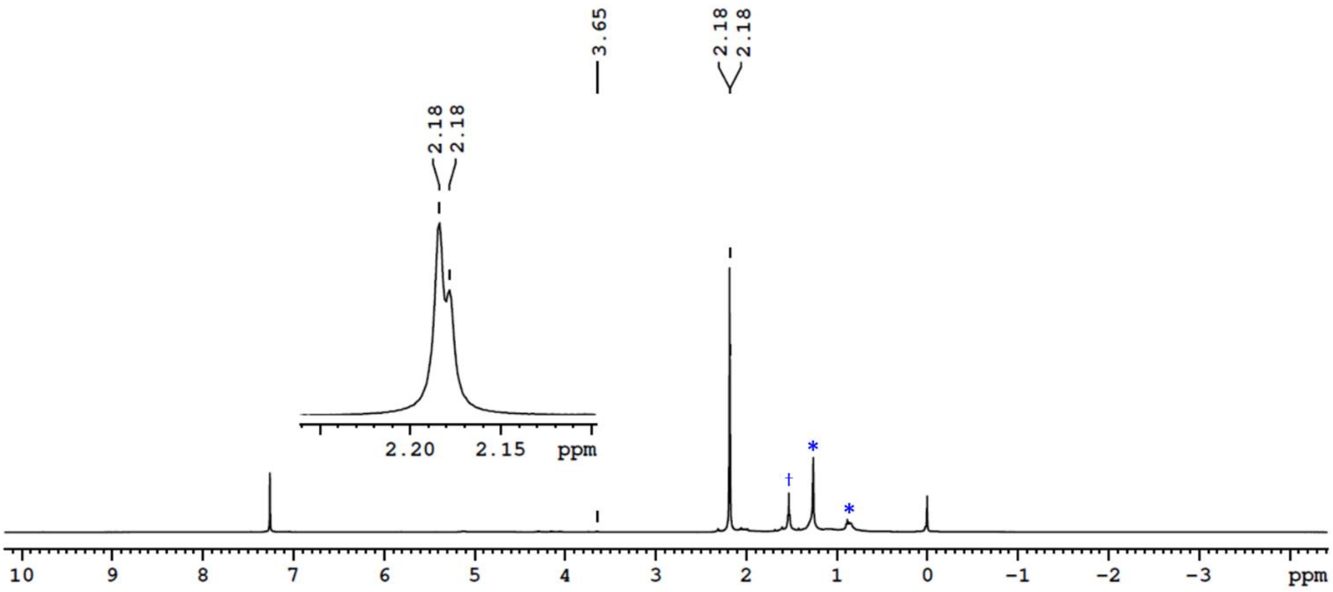

Figure S6. ^{1}H NMR spectrum of compounds **2** in $CDCl_3$. (*Grease, †H_2O)

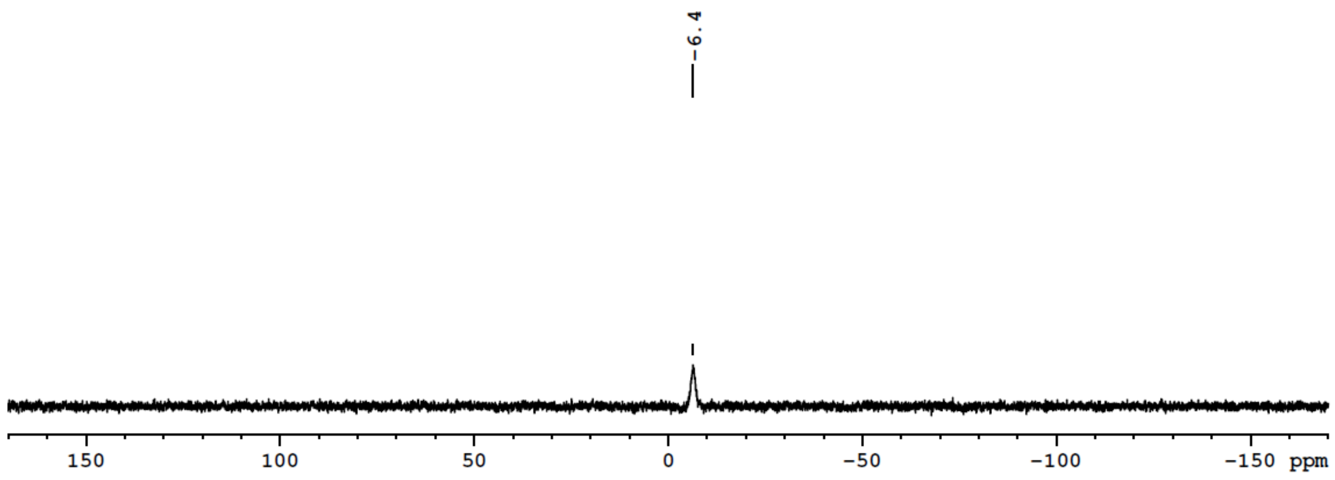

Figure S7. $^{11}B\{^1H\}$ NMR spectrum of compound **2** in $CDCl_3$ [1-4].

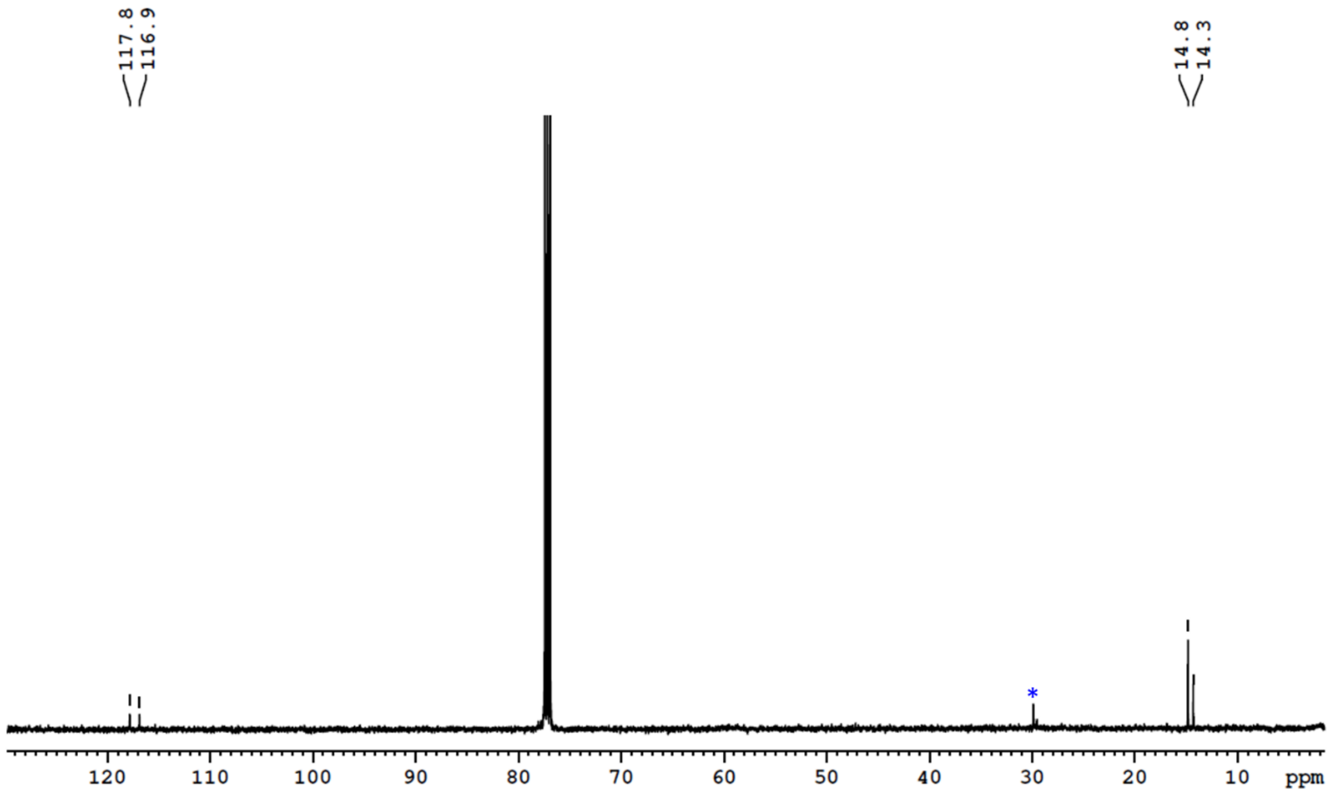

Figure S8. $^{13}C\{^1H\}$ NMR spectrum of compounds **2** in $CDCl_3$. (*Grease)

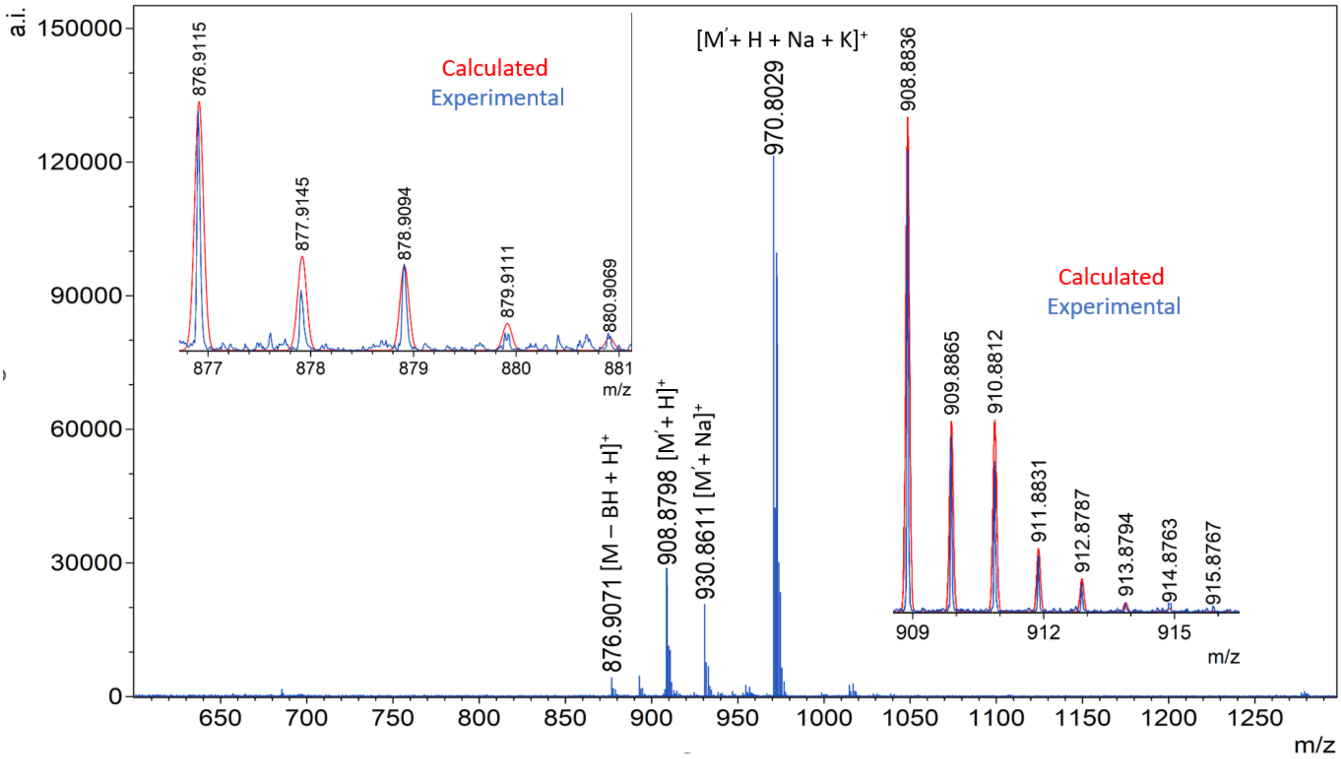

Figure S9. Combined ESI-MS of compounds **3** and **4.**

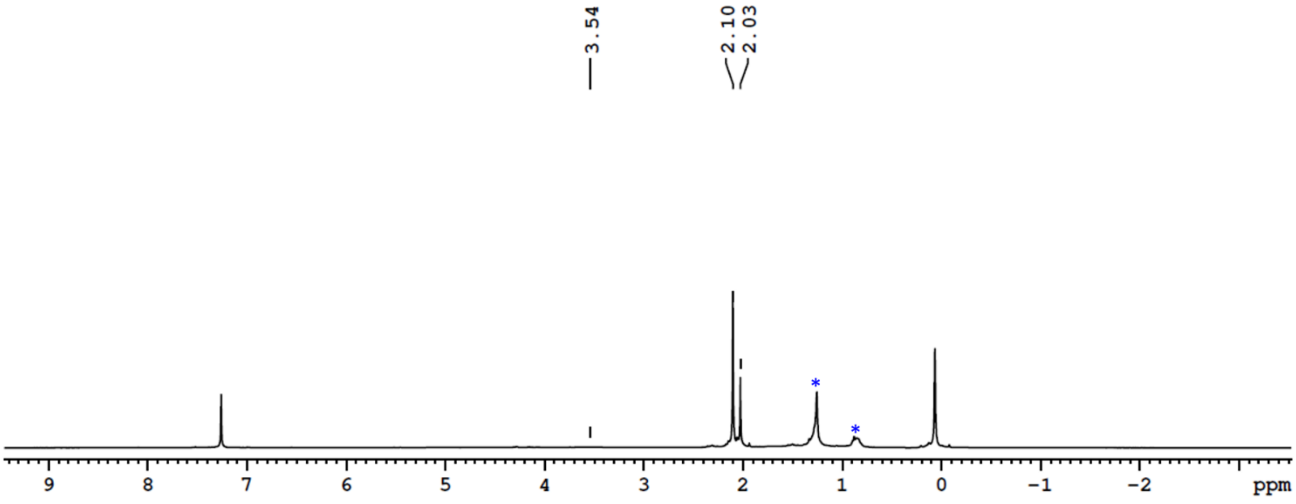

Figure S10. Combined ^{1}H NMR spectrum of compounds **3** and **4** in $CDCl_3$. (*Grease)

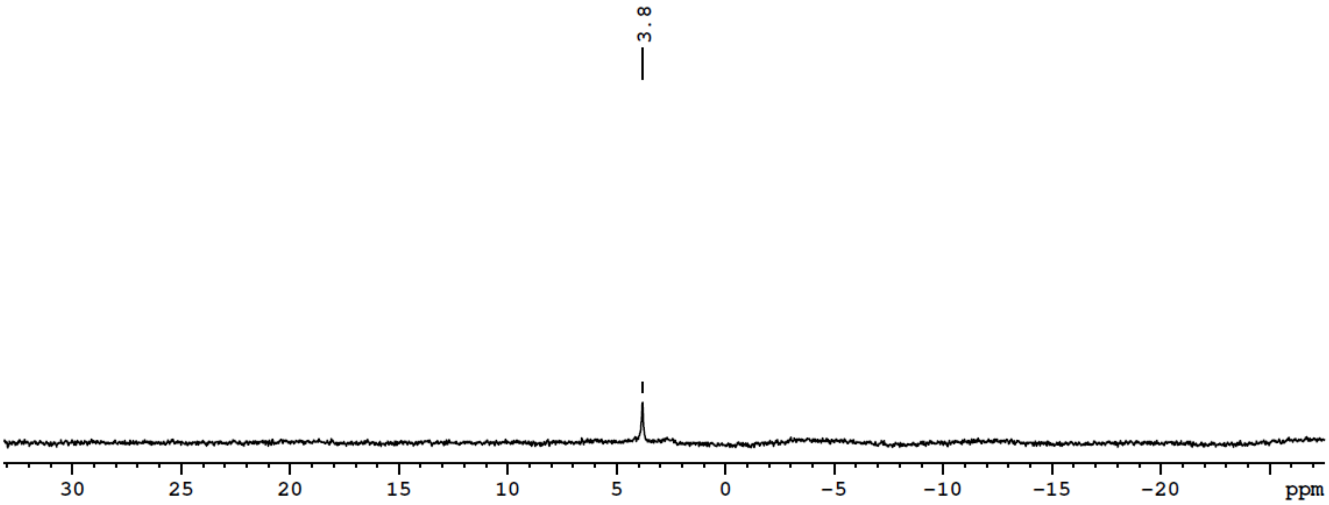

Figure S11. Combined $^{11}B\{^{1}H\}$ NMR spectrum of compounds **3** and **4** in $CDCl_3$ [1-4].

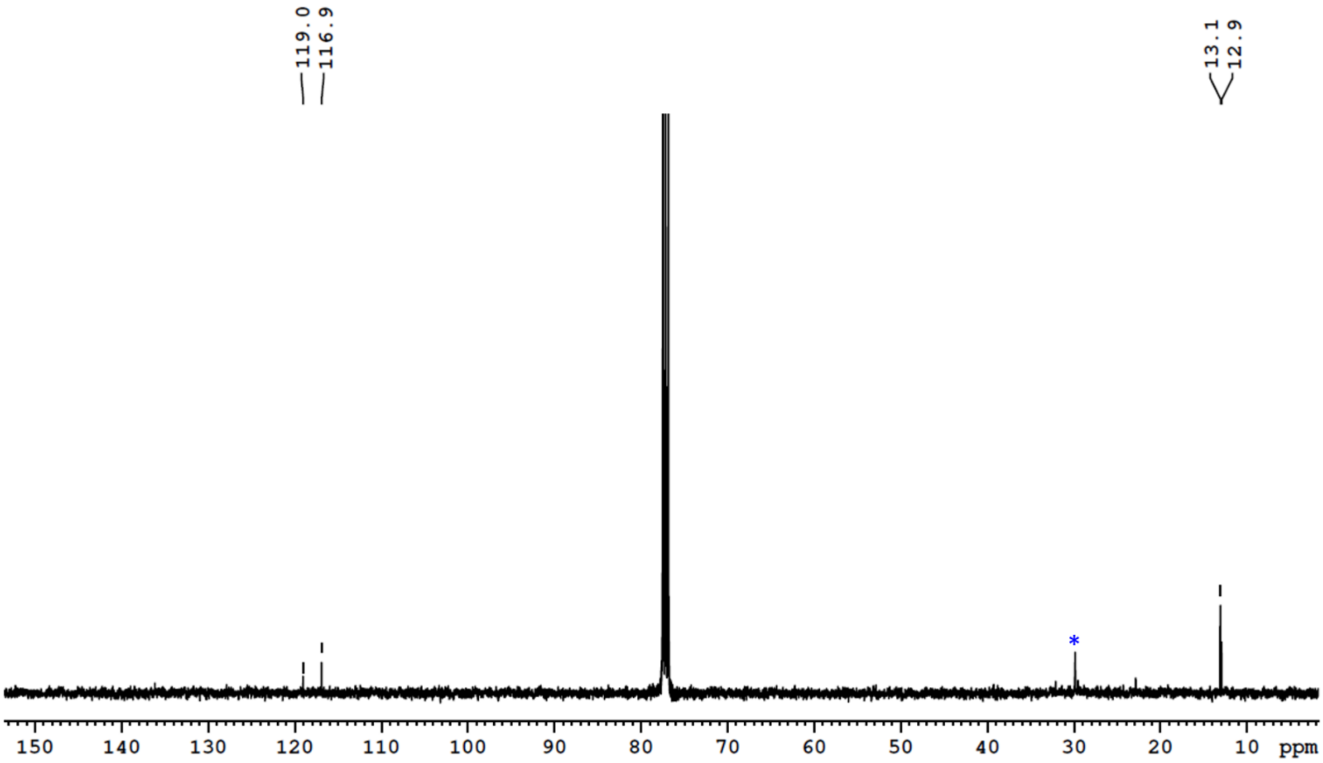

Figure S12. Combined $^{13}C\{^{1}H\}$ NMR spectrum of compounds **3** and **4** in $CDCl_3$**.** (*Grease)

II. Electronic Structure Analysis

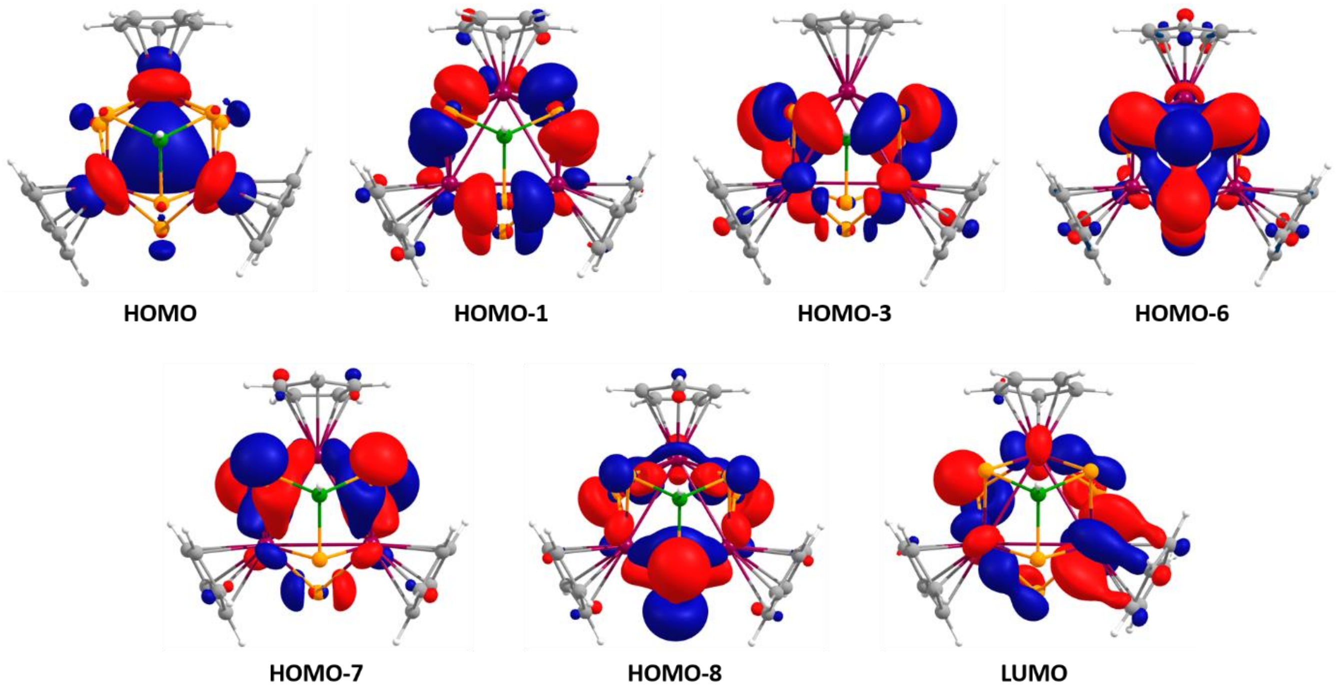

Figure S13. Selected molecular orbitals of **1**. (contour values for isosurface = ± 0.04 [e/bohr^3]$^{1/2}$).

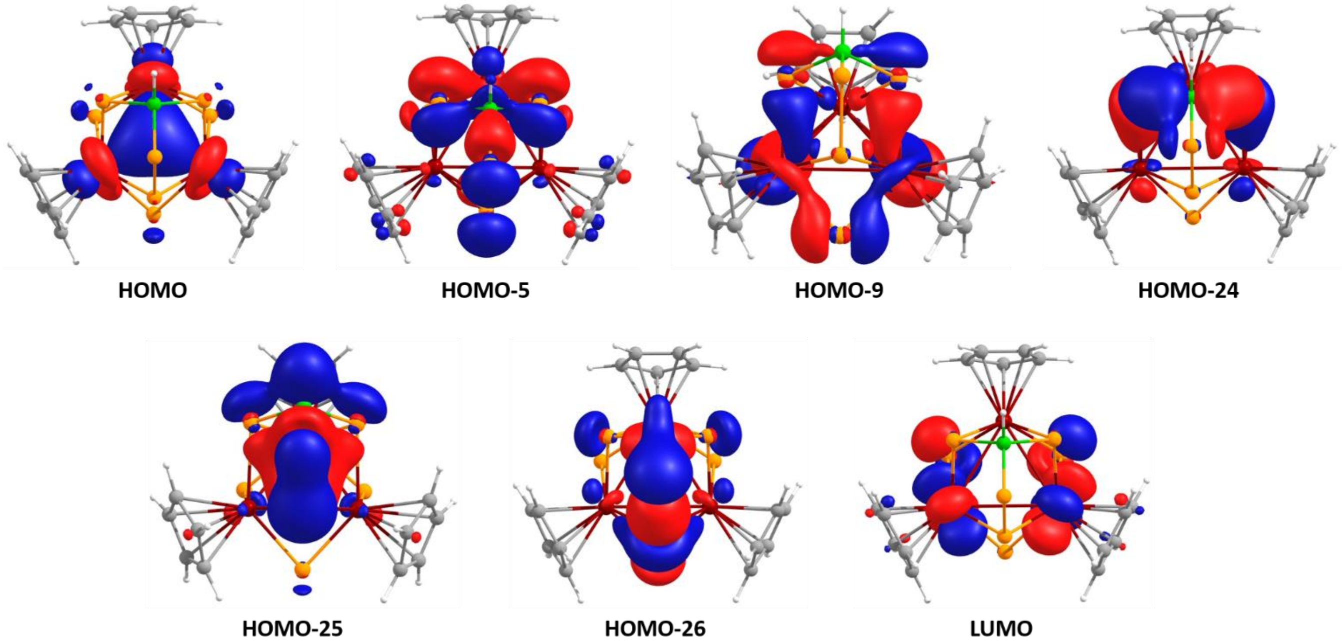

Figure S14. Selected molecular orbitals of **2**. (contour values for isosurface = ± 0.04 [e/bohr^3]$^{1/2}$).

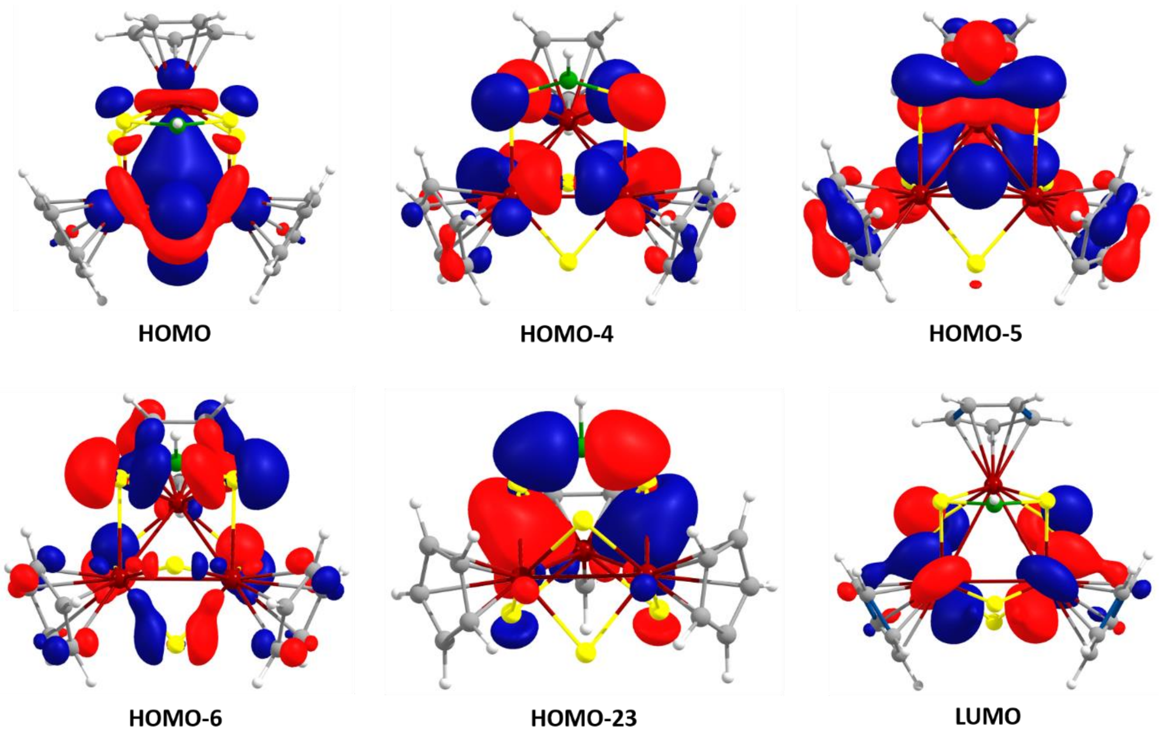

Figure S15. Selected molecular orbitals of **3**. (contour values for isosurface = ± 0.04 [e/bohr^3]$^{1/2}$).

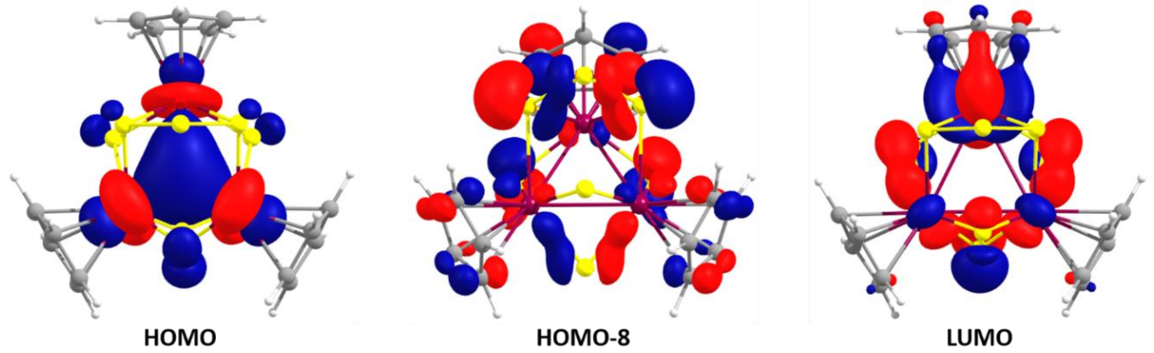

Figure S16. Selected molecular orbitals of **4.** (contour values for isosurface = ± 0.04 [e/bohr^3]$^{1/2}$).

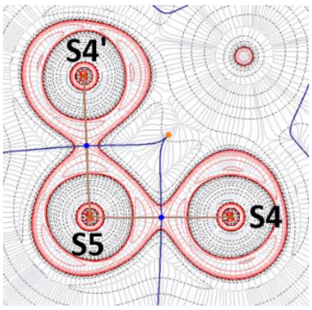

Figure S17. contour-line diagrams of the Laplacian of electron density in the S4-S5-S4' plane of **4**. In the contour-line diagrams of the Laplacian of electron density, solid red lines indicate areas of charge concentration [$\nabla^2\rho(r) < 0$], while dashed black lines show areas of charge depletion [$\nabla^2\rho(r) > 0$]. Blue dots indicate bond critical points (BCPs), and the brown line depicts the bond paths.

Table S1. NICS values in compounds **1**-**4**.

	Compound **1**	Compound **2**	Compound **3**	Compound **4**
NICS(0)	-16.8	-20.3	-17.7	-21.0
$NICS_{zz}(0)$	-48.1	-46.8	-45.7	-47.5
NICS(0.5)	-10.5	-16.1	-10.9	-19.3
$NICS_{zz}(0.5)$	-33.4	-35.5	-31.6	-36.9
NICS(-0.5)	-17.3	-19.8	-18.5	-18.5
$NICS_{zz}(-0.5)$	-44.5	-42.3	-40.7	-40.3
NICS(1)	-1.6	-10.8	-2.5	-14.6
$NICS_{zz}(1)$	-11.9	-18.4	-10.0	-19.6
NICS(-1)	-15.6	-17.4	-16.6	-15.6
$NICS_{zz}(-1)$	-32.8	-31.3	-29.3	-27.2

Table S2. Calculated natural charges (q_{Nb}, q_B and q_E), natural valence population (Pop) and HOMO–LUMO gaps of compounds **1**-**4** (E = Se or S).

Compounds	q_{Nb}	q_B	q_E	Pop (Nb_{val})	Pop (B_{val})	Pop (E_{val})	ΔE_{H-L} (eV)
**1**	-0.574	-0.155	0.209	5.510	3.122	5.776	1.296
	-0.574		0.209	5.510		5.776	
	-0.578		0.214	5.139		5.771	
			0.310			5.679	
			0.310			5.679	
			0.312			5.677	
**2**	-0.617	-0.281	-0.026	5.554	3.247	6.007	1.224
	-0.617		0.251	5.554		5.733	
	-0.560		0.251	5.496		5.733	
			0.262			5.715	
			0.323			5.665	
			0.323			5.665	
			0.315			5.673	
**3**	-0.378	0.304	-0.031	5.312	2.662	6.015	1.467
	-0.378		-0.031	5.312		6.015	
	-0.338		0.043	5.264		5.944	
			0.167			5.819	
			0.167			5.819	
			0.167			5.819	
**4**	-0.425	-	0.027	5.044	-	5.941	1.402
	-0.425		0.147			5.827	
	-0.393		0.147			5.827	
			0.144			5.842	
			0.144			5.842	
			0.173			5.813	
			0.113			5.872	

Table S3. Wiberg Bond Indices (WBI) of selected bonds in **1**-**4**.

Compound **1**		Compound **2**		Compound **3**		Compound **4**	
Bonds	WBIs	Bonds	WBIs	Bonds	WBIs	Bonds	WBIs
Nb-Nb	0.362, 0.362, 0.365	Nb-Nb	0.324, 0.381, 0.381	Nb-Nb	0.355, 0.355, 0.467	Nb-Nb	0.331, 0.331, 0.532
B-Se	0.888, 0.888, 0.883	B-Se	0.857, 0.857, 1.064	B-S	1.047, 1.047	S-S	0.956, 0.956

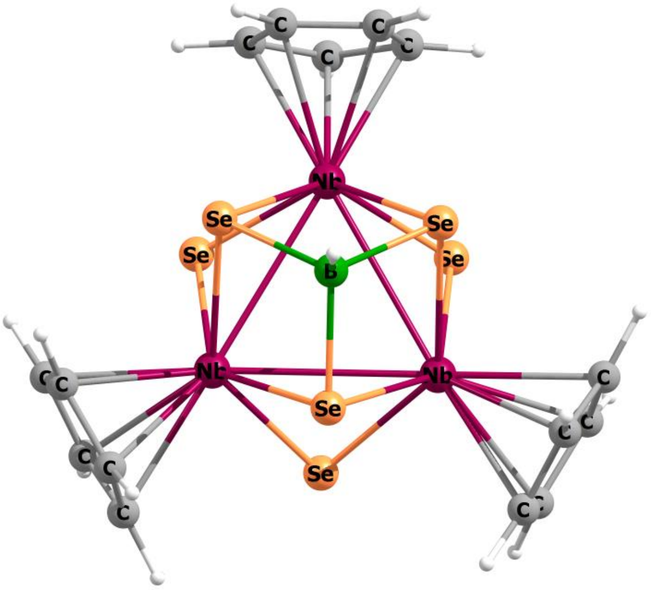

Figure S18. Optimized geometry of **1**

Total energy = -15186.64838990 a.u.

Cartesian coordinates for the calculated structure **1** (in Å)

Nb	-0.145085000000	-0.958719000000	1.651739000000
Nb	-0.148633000000	1.912749000000	0.000000000000
C	-0.505895000000	-1.016703000000	4.081052000000
C	-1.367399000000	-1.997108000000	3.491044000000
C	-0.543073000000	-3.022221000000	2.925253000000
C	0.822064000000	-2.662058000000	3.140073000000
C	0.845048000000	-1.420742000000	3.855838000000
C	-1.125459000000	4.074671000000	0.713442000000
C	0.234599000000	4.046195000000	1.155338000000
C	1.076683000000	4.031883000000	0.000000000000
Se	-1.637118000000	-2.155311000000	0.000000000000
Se	-1.617646000000	1.083481000000	1.879579000000
Se	1.779278000000	-1.874092000000	0.000000000000
Se	1.781607000000	0.932036000000	-1.613203000000
B	2.700827000000	-0.006604000000	0.000000000000
H	3.918138000000	-0.012237000000	0.000000000000
C	-1.125459000000	4.074671000000	-0.713442000000
C	0.234599000000	4.046195000000	-1.155338000000
Se	1.781607000000	0.932036000000	1.613203000000
Nb	-0.145085000000	-0.958719000000	-1.651739000000
C	-0.505895000000	-1.016703000000	-4.081052000000
C	-1.367399000000	-1.997108000000	-3.491044000000
C	-0.543073000000	-3.022221000000	-2.925253000000
C	0.822064000000	-2.662058000000	-3.140073000000
C	0.845048000000	-1.420742000000	-3.855838000000
Se	-1.617646000000	1.083481000000	-1.879579000000
H	2.173466000000	4.003007000000	0.000000000000
H	0.572807000000	4.019460000000	-2.198730000000
H	-2.011916000000	4.098750000000	-1.358596000000
H	-2.011916000000	4.098750000000	1.358596000000
H	0.572807000000	4.019460000000	2.198730000000
H	-0.826266000000	-0.111538000000	4.610266000000
H	-2.464382000000	-1.976696000000	3.491031000000
H	-0.896706000000	-3.925070000000	2.413722000000
H	1.699978000000	-3.239747000000	2.827994000000
H	1.743308000000	-0.883483000000	4.182164000000
H	1.699978000000	-3.239747000000	-2.827994000000
H	-0.896706000000	-3.925070000000	-2.413722000000
H	-2.464382000000	-1.976696000000	-3.491031000000
H	-0.826266000000	-0.111538000000	-4.610266000000
H	1.743308000000	-0.883483000000	-4.182164000000

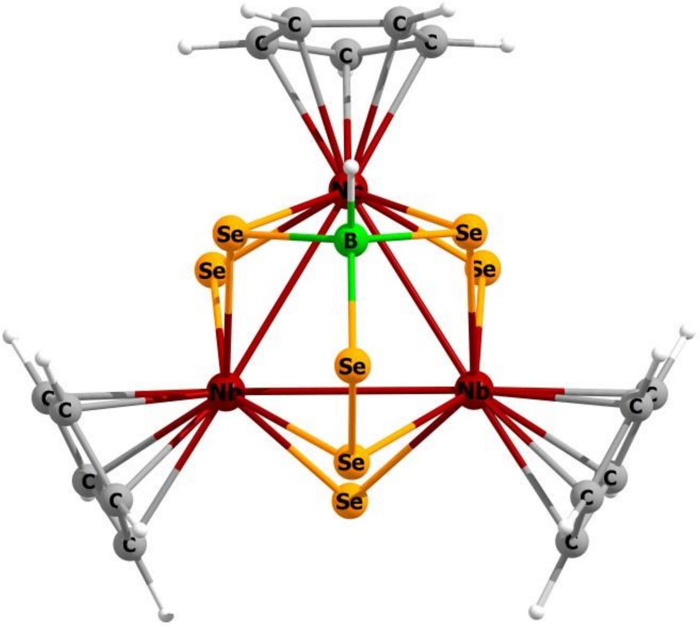

Figure S19. Optimized geometry of **2**

Total energy = -17588.21549380 a.u.

Cartesian coordinates for the calculated structure **2** (in Å)

B	-0.220851000000	0.000056000000	2.847468000000	H	4.280185000000	-2.201229000000	0.212176000000
C	-2.551212000000	3.215959000000	-0.225598000000	H	3.474943000000	-1.360409000000	2.667229000000
C	-1.368782000000	3.901464000000	0.200233000000	H	3.475098000000	1.360757000000	2.666745000000
C	-0.551252000000	4.124491000000	-0.949624000000	H	0.421238000000	4.630689000000	-0.958655000000
C	-1.224200000000	3.572978000000	-2.087914000000	H	-1.140108000000	4.209386000000	1.227309000000
C	-2.466085000000	3.023879000000	-1.639604000000	H	-3.380838000000	2.907730000000	0.421760000000
C	-1.225680000000	-3.572361000000	-2.088089000000	H	-3.220132000000	2.534055000000	-2.267176000000
C	-2.467196000000	-3.023294000000	-1.638710000000	H	-0.856453000000	3.581224000000	-3.121817000000
C	-2.551327000000	-3.215823000000	-0.224702000000	H	-3.221628000000	-2.533162000000	-2.265578000000
Nb	-0.656580000000	1.697791000000	-0.621555000000	H	-3.380457000000	-2.907719000000	0.423355000000
Nb	-0.656906000000	-1.697671000000	-0.621529000000	H	-0.858668000000	-3.580303000000	-3.122256000000
C	-0.551975000000	-4.124323000000	-0.950466000000	H	0.420436000000	-4.630656000000	-0.960362000000
C	-1.368658000000	-3.901589000000	0.200046000000	H	-1.139270000000	-4.209867000000	1.226857000000
C	3.735682000000	0.716973000000	1.818443000000	H	0.303903000000	0.000023000000	3.951861000000
C	4.163254000000	1.157165000000	0.527481000000	Se	-2.231259000000	0.000262000000	2.948377000000
C	4.411259000000	-0.000402000000	-0.278354000000	Se	0.499125000000	1.629662000000	1.759578000000
C	4.163141000000	-1.157653000000	0.527893000000	Se	0.498804000000	-1.629728000000	1.759634000000
C	3.735603000000	-0.716956000000	1.818698000000	Se	-2.407926000000	0.000233000000	0.552744000000
Nb	2.023121000000	-0.000245000000	0.218416000000	Se	1.714521000000	1.850121000000	-1.469242000000
H	4.280434000000	2.200615000000	0.211401000000	Se	-1.419742000000	0.000067000000	-2.354773000000
H	4.750245000000	-0.000603000000	-1.321876000000	Se	1.714267000000	-1.850580000000	-1.469123000000

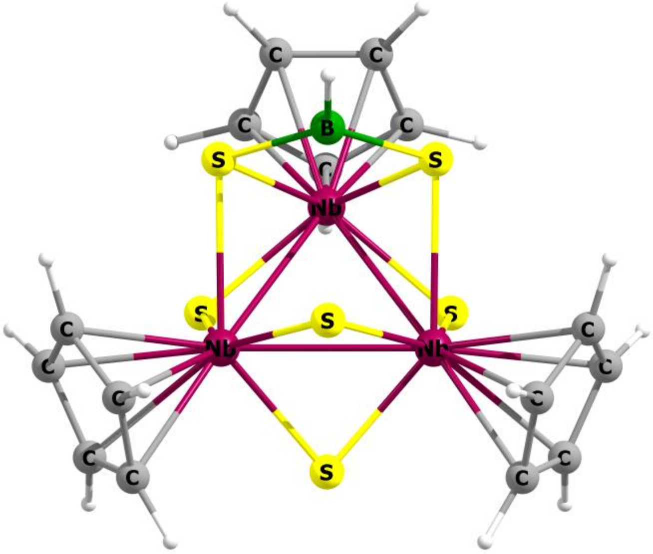

Figure S20. Optimized geometry of **3.**

Total energy = -3166.07166899 a.u.

Cartesian coordinates for the calculated structure **3** (in Å)

Nb	0.987690000000	1.512585000000	-0.052489000000	C	2.783889000000	-2.970737000000	0.778416000000
Nb	-1.888828000000	-0.000347000000	-0.123699000000	C	1.564713000000	-3.703743000000	0.930725000000
C	-4.005482000000	1.158159000000	-0.611220000000	C	1.039252000000	-3.965283000000	-0.372708000000
C	-4.088161000000	0.715464000000	0.742405000000	C	1.921951000000	-3.380750000000	-1.333342000000
C	-4.088022000000	-0.717835000000	0.741426000000	S	1.822649000000	0.000204000000	-1.731168000000
C	-4.005279000000	-1.158664000000	-0.612801000000	H	3.433765000000	-2.623285000000	1.589725000000
C	-3.934673000000	0.000322000000	-1.451624000000	H	1.109228000000	-4.005412000000	1.882194000000
C	1.563294000000	3.704297000000	0.930809000000	H	0.108413000000	-4.500321000000	-0.595870000000
C	1.037471000000	3.965611000000	-0.372528000000	H	1.791627000000	-3.391729000000	-2.422552000000
C	1.920349000000	3.381621000000	-1.333335000000	H	3.849449000000	-2.234425000000	-1.068837000000
S	-0.991616000000	-1.544717000000	1.766885000000	H	3.432995000000	2.624789000000	1.589468000000
S	-1.028295000000	-1.780093000000	-1.416317000000	H	3.848528000000	2.236351000000	-1.069186000000
S	-1.028791000000	1.779256000000	-1.416667000000	H	1.107803000000	4.005657000000	1.882374000000
S	2.025873000000	0.000403000000	1.578964000000	H	0.106273000000	4.500083000000	-0.595541000000
C	2.782824000000	2.971943000000	0.778264000000	H	1.789843000000	3.392595000000	-2.422524000000
C	3.002505000000	2.770889000000	-0.620961000000	H	-3.980083000000	2.201121000000	-0.947761000000
S	-0.992236000000	1.544372000000	1.766650000000	H	-3.848590000000	0.001098000000	-2.545366000000
B	-0.685711000000	-0.000056000000	2.730005000000	H	-3.979740000000	-2.201172000000	-0.950735000000
H	-0.544920000000	0.000108000000	3.935052000000	H	-4.135670000000	-1.363142000000	1.627077000000
Nb	0.988075000000	-1.512319000000	-0.052512000000	H	-4.135972000000	1.359547000000	1.628940000000
C	3.003677000000	-2.769475000000	-0.620752000000				

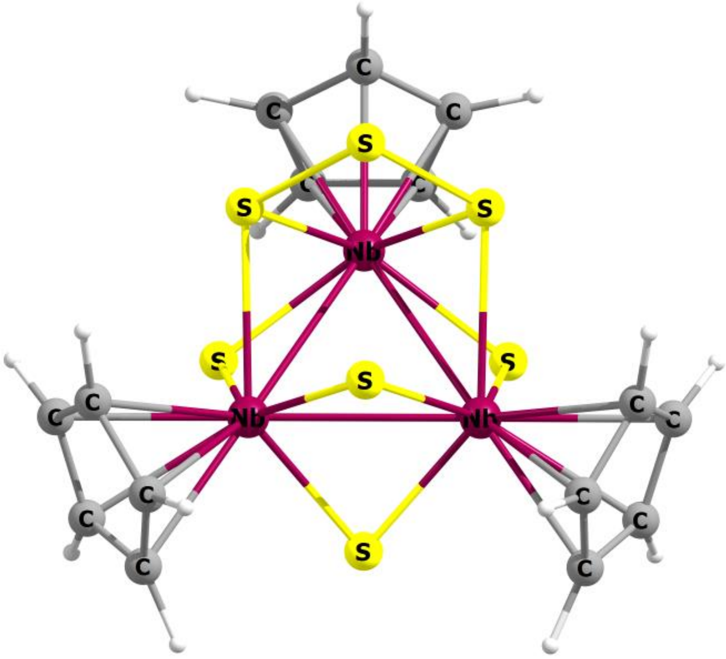

Figure S21. Optimized geometry of **4.**

Total energy = -3538.73407560 a.u.

Cartesian coordinates for the calculated structure **4** (in Å)

Nb	1.878372000000	-0.000032000000	-0.302178000000
Nb	-1.052999000000	1.491642000000	-0.148786000000
C	-1.114935000000	3.962996000000	-0.080373000000
C	-1.822786000000	3.489256000000	1.067531000000
C	-2.986217000000	2.793708000000	0.608415000000
C	-2.992014000000	2.834956000000	-0.822658000000
C	-1.833847000000	3.556852000000	-1.248752000000
C	4.074769000000	0.000147000000	0.765946000000
C	4.038015000000	1.156788000000	-0.075276000000
C	3.965295000000	0.714488000000	-1.431280000000
S	-1.863574000000	0.000100000000	1.563353000000
S	-1.883506000000	-0.000041000000	-1.833128000000
S	0.996949000000	1.861568000000	-1.463419000000
S	1.038933000000	-1.543687000000	1.553962000000
S	0.883977000000	0.000025000000	3.024685000000
C	4.038016000000	-1.156782000000	-0.074883000000
C	3.965304000000	-0.714945000000	-1.431036000000
S	1.038969000000	1.543694000000	1.553915000000
Nb	-1.053046000000	-1.491620000000	-0.148730000000
C	-1.115069000000	-3.963082000000	-0.082915000000
C	-1.820902000000	-3.490592000000	1.066755000000
C	-2.985074000000	-2.794448000000	0.610455000000
C	-2.993300000000	-2.833982000000	-0.820650000000
C	-1.835895000000	-3.555461000000	-1.249594000000
S	0.996867000000	-1.861581000000	-1.463412000000
H	3.903453000000	1.359064000000	-2.316610000000
H	4.026767000000	2.199759000000	0.263861000000
H	4.106253000000	0.000331000000	1.863397000000
H	4.026772000000	-2.199638000000	0.264608000000
H	3.903469000000	-1.359822000000	-2.316147000000
H	-0.170985000000	4.522238000000	-0.070191000000
H	-1.526329000000	3.626738000000	2.114741000000
H	-3.738442000000	2.309047000000	1.241603000000
H	-3.752591000000	2.392187000000	-1.476627000000
H	-1.540464000000	3.750205000000	-2.288011000000
H	-3.754899000000	-2.390269000000	-1.472787000000
H	-3.736259000000	-2.310626000000	1.245513000000
H	-1.522702000000	-3.629387000000	2.113297000000
H	-0.171188000000	-4.522463000000	-0.075010000000
H	-1.544288000000	-3.747582000000	-2.2895850000
